# Spatial and Temporal Distribution of Dopaminergic Neurons during Development in Zebrafish

**DOI:** 10.3389/fnana.2016.00115

**Published:** 2016-11-28

**Authors:** Yuchen Du, Qiang Guo, Minghui Shan, Yongmei Wu, Sizhou Huang, Haixia Zhao, Huarong Hong, Ming Yang, Xi Yang, Liyi Ren, Jiali Peng, Jing Sun, Hongli Zhou, Shurong Li, Bingyin Su

**Affiliations:** ^1^Development and Regeneration Key Lab of Sichuan Province, Department of Anatomy and Histology and Embryology, Department of Pathology, Chengdu Medical CollegeChengdu, China; ^2^Chongqing Key Laboratory of Neurobiology, Department of Neurobiology, Third Military Medical UniversityChongqing, China; ^3^Chengdu Medical College Infertility HospitalChengdu, China

**Keywords:** Parkinson’s disease, dopamine, development, distribution, projection, zebrafish

## Abstract

As one of the model organisms of Parkinson’s disease (PD) research, the zebrafish has its advantages, such as the 87% homology with human genome and transparent embryos which make it possible to observe the development of dopaminergic neurons in real time. However, there is no midbrain dopaminergic system in zebrafish when compared with mammals, and the location and projection of the dopaminergic neurons are seldom reported. In this study, Vmat2:GFP transgenic zebrafish was used to observe the development and distribution of dopaminergic neurons in real time. We found that diencephalons (DC) 2 and DC4 neuronal populations were detected at 24 h post fertilization (hpf). All DC neuronal populations as well as those in locus coeruleus (LC), raphe nuclei (Ra) and telencephalon were detected at 48 hpf. Axons were detected at 72 hpf. At 96 hpf, all the neuronal populations were detected. For the first time we reported axons from the posterior tubercle (PT) of ventral DC projected to subpallium *in vivo*. However, when compared with results from whole mount tyrosine hydroxylase (TH) immunofluorescence staining in wild type (WT) zebrafish, we found that DC2 and DC4 neuronal populations were mainly dopaminergic, while DC1, DC3, DC5 and DC6 might not. Neurons in pretectum (Pr) and telencephalon were mainly dopaminergic, while neurons in LC and Ra might be noradrenergic. Our study makes some corrections and modifications on the development, localization and distribution of zebrafish dopaminergic neurons, and provides some experimental evidences for the construction of the zebrafish PD model.

## Introduction

Parkinson’s disease (PD) is a neurodegenerative disease mainly caused by degeneration and death of dopaminergic neurons in substantia nigra pars compacta and the resulting dopamine (DA) deficiency in corpus striatum. Among model organisms used for PD research, zebrafish has its advantages of the 87% homology with human genome (Ma, 1994), small volume and transparent embryos and early larvae which make it possible to observe the complete central nervous system during embryonic period. Study on the development of zebrafish dopaminergic nervous system will be helpful for a more accurate understanding of the vertebrate dopaminergic neurons and their axonal projections (Ma, 1994, 1997; Smeets and González, 2000).

In mammalian model, the dopaminergic system is mainly distributed in the substantia nigra and the ventral tegmental area. Zebrafish does not has a dopaminergic system, and the tyrosine hydroxylase (TH) immunopositive neurons are considered to be dopaminergic neurons (Kaslin and Panula, 2001), whose development, distribution and axonal projection have not been reported systematically. In addition, for most animals such as mice and rats, the observation of neurons can only be made through successive sections and immunohistochemical staining (Blechman et al., 2007). These methods can reflect the general changes of neurons and axons, but not the dynamic real-time development of them (Thirumalai and Cline, 2008). The embryos and early larvae of Vmat2:GFP transgenic zebrafish are transparent and can be observed *in vivo* and therefore have been used in the developmental research on the nervous system, cardiovascular system and immune system in recent years (Riparbelli and Callaini, 2007; Yang et al., 2008). In this study, the Vmat2:GFP zebrafish and TH whole mount immunofluorescent staining were used to detect the dominergic neurons from fertilized eggs to the completely developed process and their distribution and axonal projection. Our study made some corrections and modifications on the development, localization and distribution of zebrafish dominergic neurons, and therefore, provided some experimental evidences for the construction of the retrograde degeneration model of zebrafish dominergic neurons. These findings have implications for exploring the pathogenesis of PD.

## Materials and Methods

### Zebrafish Embryos Photographed by Laser Scanning Confocal Microscope (LSCM)

This study was carried out in accordance with the medical ethics committee of Chengdu Medical College. All experiments were approved by the medical ethics committee of Chengdu Medical College. Vmat2:GFP embryos (*n* = 35) developed up to 24 h with fluorescent were collected and cultured in dishes with 1-phenyl 2-thiourea (PTU) to inhibit the formation of melanin. Observation was performed when embryos were anesthetized (Tricaine, 0.4%) and covered with low melting point agarose. The part between two eyes was explosed and photographed by laser scanning confocal microscope (LSCM; Olympus FV-1000).

### Whole Mount Immunofluorescent Staining

Wild type (WT) embryos (*n* = 25) were washed and fixed with 1 mL formaldehyde (4%) overnight. After 3–5 washes with PBS, the samples were blocked with 1 mL PBTN (PBS + 0.3% TritonX-100 + 4% BSA + 0.02% NaN3) at 4°C for 1 h followed by rabbit-anti-TH antibody (1:200, Sigma) application at 4°C overnight. Then the fluorescent labeled goat-anti-rabbit antibody (1:1000, Life Technologies) was applied at 4°C overnight in dark. After that, embryos were washed five times with posterior tubercle (PT) (PBS + 0.3% TritonX-100) for about 40 min each time. Observation was then performed under LSCM.

## Results

### Development of Dopaminergic Neurons in Vmat2:GFP Zebrafish

**Figure 1:**
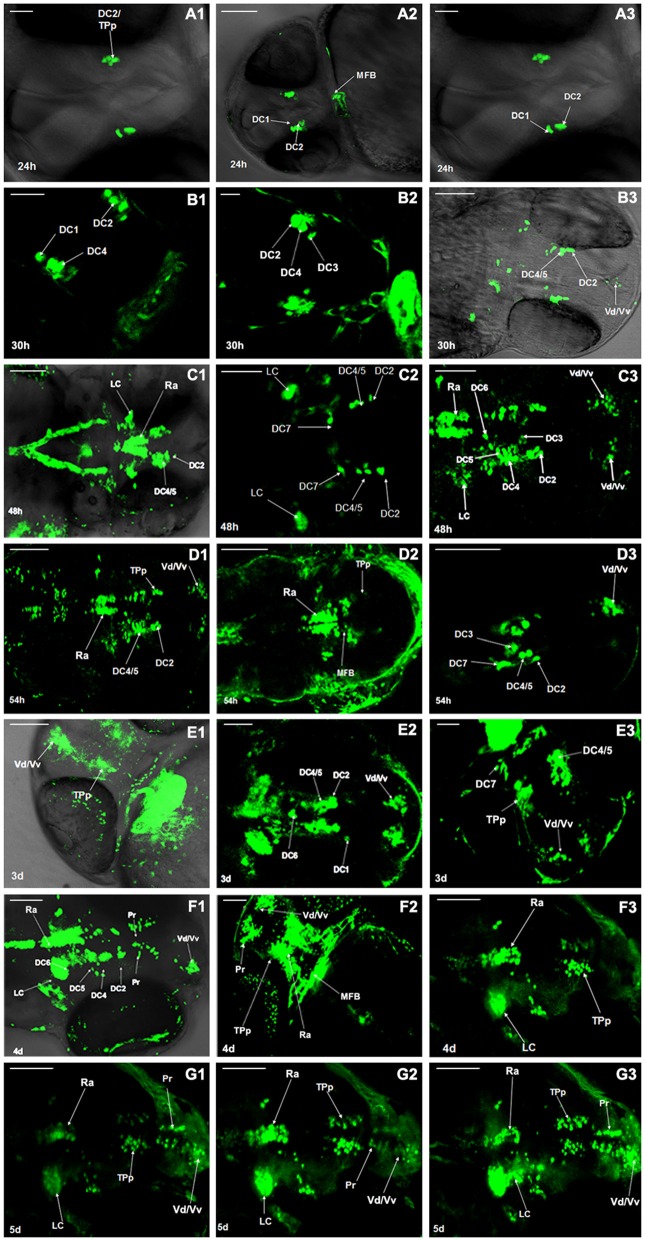
**Development of dopaminergic neurons in Vmat2:GFP zebrafish.** The development of dopaminergic neurons in ventral telencephalic area was observed repeatedly at 24 h post fertilization (hpf; **A1–A3**), 30 hpf **(B1–B3)**, 48 hpf **(C1–C3)**, 54 hpf **(D1–D3)**, 3 days post fertilization (dpf; **E1–E3**), 4 dpf **(F1–F3)**, 5 dpf **(G1–G3)**. Similar observations are demonstrated in **(A1–G3).** DC, diencephalons; TPp, periventricular nucleus of posterior tubercle; MFB, midbrain/forebrain boundary; Vd/Vv, dorsal/ventral nucleus of the telencephalic area; LC, locus coeruleus; Ra, raphe nuclei; Pr, pretectum. Scale: **(A1,A3)** = 30 μm; **(E2,E3)** = 50 μm; **(A2,B–D,E1,F,G)** = 100 μm.

Development of dopaminergic neurons in ventral telencephalic area was observed at 24 h post fertilization (hpf), 30 hpf, 48 hpf, 54 hpf, 3 days post fertilization (dpf), 4 dpf, 5 dpf, respectively. At 24 hpf the fluorescence labeling was detected in the periventricular nucleus of posterior tubercle (TPp). As the zebrafish was not fully developed at this time, we speculated the cell type to be the diencephalons (DC) 1/2 (Figures 1A1–A3). At 30 hpf, the dopaminergic neurons in PT began to differentiate into different neuronal populations and the DC2/3/4, the dorsal/ventral nucleus of the telencephalic area (Vd/Vv) were detected (Figure 1B). At 48 hpf, all the neuronal populations (DC1–7) in the ventral DC were detected, as well as those in locus coeruleus (LC), raphe nuclei (Ra), and telencephalon (Figure 1C). At 54 hpf, the neurons further differentiated and there was no evident variation in morphological characteristics (Figure 1D). At 3 dpf, axons projected from the DC2 of TPp to telencephalon were detected (Figure 1E). All the neuronal populations developed and the pretectum (Pr) were detected between TPp and Vd/Vv (Figure 1F). All the catecholaminergic neurons in the brain of Vmat2:GFP transgenic zebrafish showed fluorescence (Figure 1G).

### Development of Dopaminergic Neurons in WT Zebrafish

**Figure 2:**
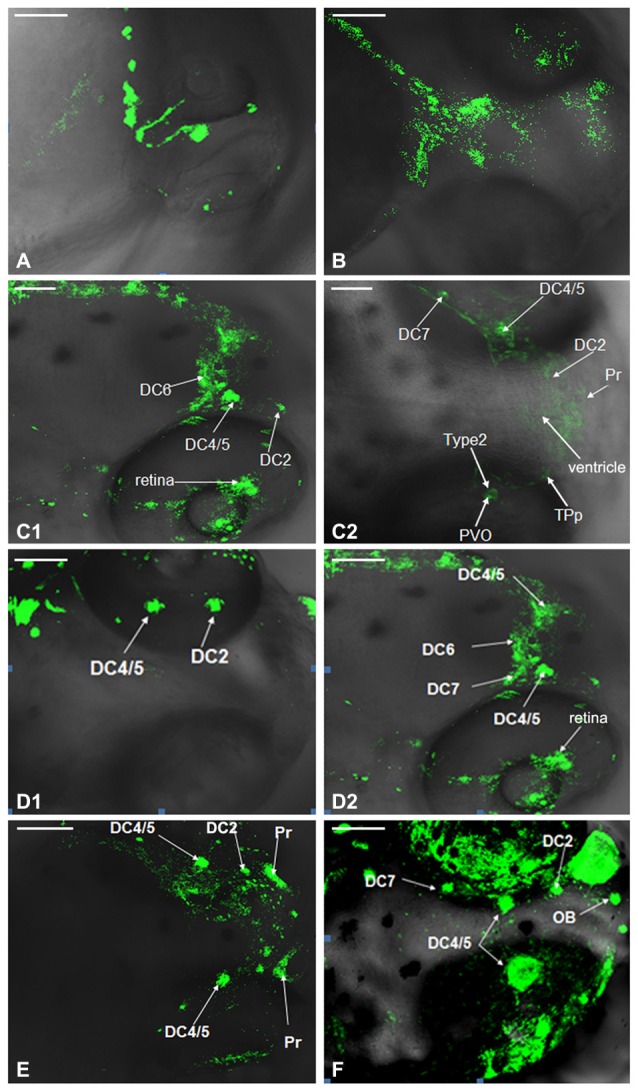
**Development of dopaminergic neurons in wild type (WT) zebrafish.** Development of dopaminergic neruons in WT zebrafish was determined by tyrosine hydroxylase (TH) whole mount immunofluorescent at 24 hpf **(A)**, 48 hpf **(B)**, 3 dpf **(C1,C2)**, 4 dpf **(D1,D2)**, 5 dpf **(E)** and 6 dpf **(F)**, respectively. In order to show that it was not an individual case, we provided **(C1,C2)**, and there was no difference between them. It was the same with **(D1,D2)**. DC, diencephalons; Pr, pretectum; TPp, periventricular nucleus of posterior tubercle; PVO, paraventricular organ; OB, olfactory bulb. Scale = 100 μm.

Development of dopaminergic neruons in WT zebrafish was observed by TH whole mount immunofluorescent at 24 hpf, 48 hpf, 3 dpf, 4 dpf, 5 dpf and 6 dpf respectively. The posterior nodule of the ventral DC was found to develop first. At 24 hpf, the DC1 and DC2/4 neuronal populations in PT were detected. The DC2/4 population may be located in the coracoid of PT and its axons project to the deuterencephalon (Figure 2A). At 48 hpf, the TH immune positive cells were detected in DC2/4/5 populations and Vd/Vv, and DC2 and DC4 populations gradually separated with the development of brain (Figure 2B). At 3 dpf, TH immune positive cells were detected in PT and hypothalamus, presumably to be the DC6 population (Figures 2C1,C2). At 4 dpf, the TH immune positive cells located in caudate nucleus of hypothalamus were DC7 population (Figures 2D1,D2). At 5 dpf, almost all the neurons were labeled by fluorescence and DC2, DC4/5, Pr were detected (Figure 2E). At 6 dpf all the CA neuronal populations had differentiated obviously (Figure 2F).

### Comparison of Dopaminergic Neuron Distribution in the Vmat2:GFP and WT Zebrafish

**Figure 3:**
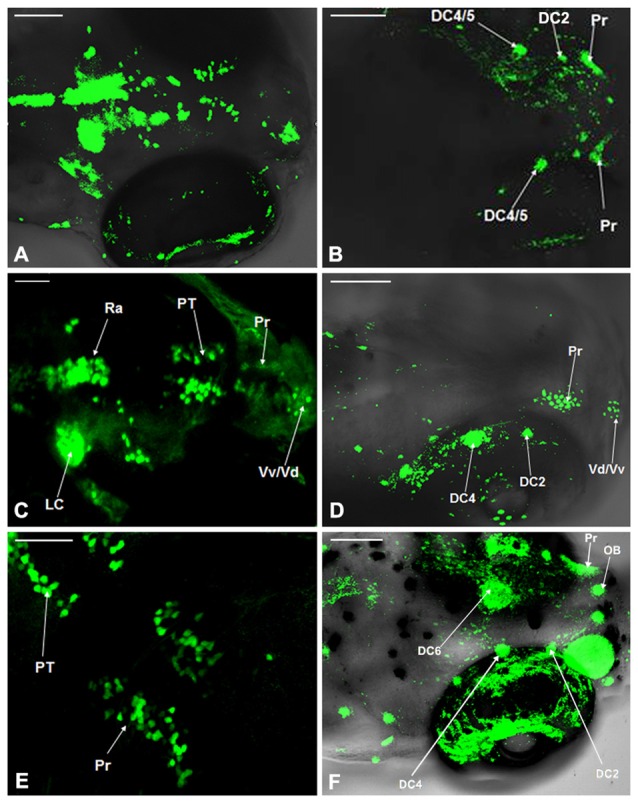
**Comparison of dopaminergic neuron distribution in the Vmat2:GFP and WT zebrafish.** The distribution of dopaminergic neurons in WT zebrafish at 4 dpf **(A)**, 5 dpf **(C)** and 6 dpf **(E)** was detected using TH whole mount immunofluorescent and compared with that in Vmat2:GFP transgenic zebrafish at 4 dpf **(B)**, 5 dpf **(D)** and 6 dpf **(F)** correspondingly. DC, diencephalons; Pr, pretectum; PT, posterior tubercle; LC, locus coeruleus; Vd/Vv, dorsal/ventral nucleus of the telencephalic area; Ra, raphe nuclei; OB, olfactory bulb. Scale: **(A,B,D,F)** = 100 μm; **(C,E)** = 50 μm.

The distribution of dopaminergic neurons in WT zebrafish at 4 dpf, 5 dpf and 6 dpf was determined using TH whole mount immunofluorescent (Figures 3A,C,E) and compared with that in Vmat2:GFP transgenic zebrafishat at matched time points (Figures 3B,D,F). At 4 dpf, all the dopaminergic neurons in the ventral DC as well as those in Vd/Vv, Pr, DC and Ra were detected in Vmat2:GFP zebrafish (Figure 3B). However, in WT zebrafish, the TH immune positive cells were detected only in DC2, DC4/5 and Pr. The nucleus diameter was about 20 μm, located on both sides of the eyes and distributed symmetrically (Figure 3A). At 5 dpf, the fluorescent label was detected in the telencephalon and Pr in both WT (Figure 3C) and Vmat:GFP zebrafish (Figure 3D), while only DC2 and DC4 were identified in the ventral DC of WT zebrafish (Figure 3C). At 6 dpf, DC2/4/6 in the ventral DC and the olfactory bulb (OB) were detected in Vmat2:GFP zebrafish (Figure 3F). The OB located symmetrically in front of the telencephalon and its diameter was about 10 μm.

### Localization of Dopaminergic Nuclei in Vmat2:GFP Zebrafish

**Figure 4:**
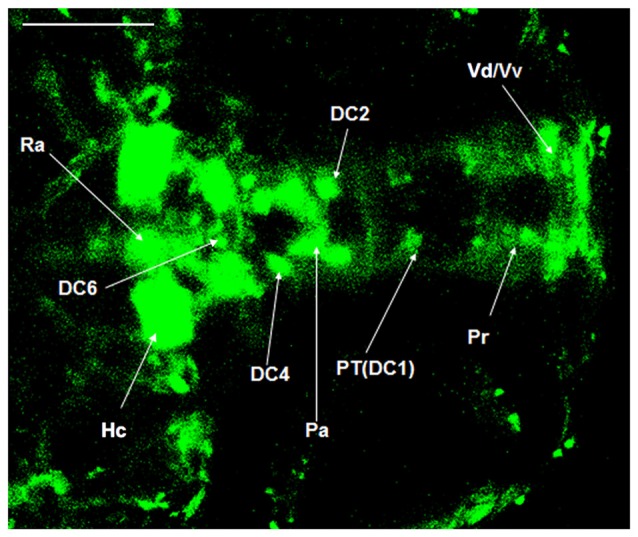
**Localization of dopaminergic nuclei in Vmat2:GFP zebrafish.** The dopaminergic nuclei in the ventral DC of the Vmat2:GFP zebrafish at 4 dpf were identified under laser scanning confocal microscope (LSCM). All the dopaminergic neurons had already been developed. DC, diencephalons; Pa, paraventricular nucleus; Pr, pretectum; PT, posterior tubercle; Vd/Vv, dorsal/ventral nucleus of the telencephalic area; Ra, raphe nuclei; Hc, caudal hypothalamus. Scale = 100 μm.

The dopaminergic nuclei in the ventral DC of the Vmat2:GFP zebrafish at 4 dpf were evaluated under LSCM. All the dopaminergic neurons were developed when the examination was conducted. They located in the Vd/Vv and ventral DC. The round DC1 neuronal population was located in the coracoid of PT, the anterior hypothalamus. The DC2 was located in the center of the anterior part of PT, near the hypothalamic paraventricular nucleus (PVN). The DC3 was located in PVN (Pa). The DC4 was located in the center of the posterior part of PT. The DC5 and DC6 located in the hypothalamus and the center part of PT. There was a gap between DC2 and DC4 (Figure 4).

### Morphology Evaluation of Dopaminergic Neurons in Vmat2:GFP Zebrafish

**Figure 5:**
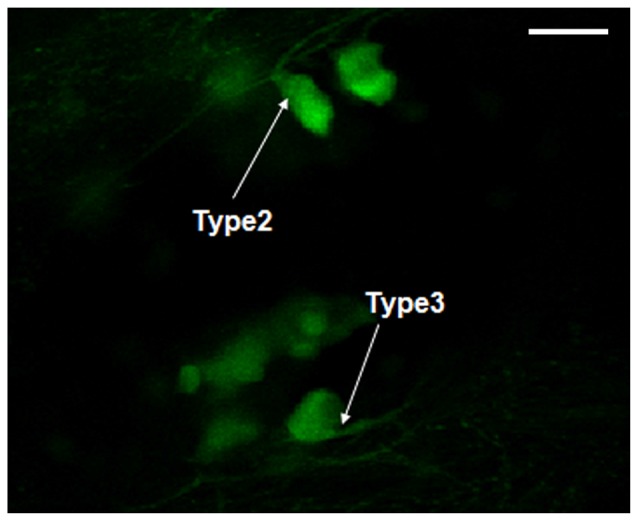
**Morphology observation of dopaminergic neurons in Vmat2:GFP zebrafish.** According to the classification of dopaminergic neurons (Kawakami et al., 2000), the observed dopaminergic neurons were mainly Type 2 and Type 3. Scale = 50 μm.

The morphology of dopaminergic neurons, mainly in DC2 and DC4 cell populations, was observed under oil lens. According to the classification of dopaminergic neurons (Kawakami et al., 2000), we observed mainly Type 2 and Type 3. The Type 1 neurons were not found (Figure 5).

### Axonal Projection of Dopaminergic Neurons in Vmat2:GFP Zebrafish

**Figure 6:**
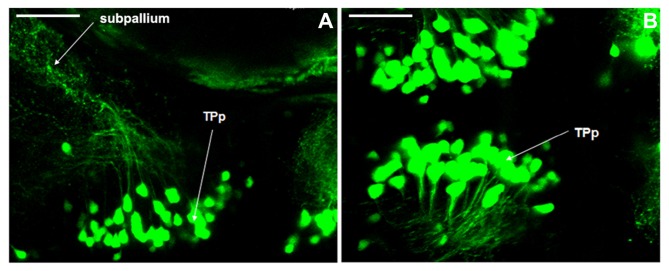
**Axonal projections of dopaminergic neurons in Vmat2:GFP zebrafish.** The axonal projections from periventricular nucleus of posterior tubercle (TPp) to subpallium **(A,B)** were found at 5 dpf. TPp, periventricular nucleus of posterior tubercle. Scale = 40 μm.

We have discovered that axons in DC2 projected to the telencephalon at 5 dpf, while the axonal projections in other neuronal populations were not clear. It was hypothesized that axons in TPp project to the subpallium (Kawakami et al., 2000). In this study, for the first time, we observed clearly the axonal projections from TPp to subpallium (Figures 6A,B), and therefore, proved this hypothesis.

## Discussion

The ventral DCs, where the majority of neurons are dopaminergic, is considered as the substantia nigra in zebrafish, where the mainly neurons distributed in are dopaminergic. These neurons can be divided into seven cell populations, that is, DC1, DC2, DC3, DC4, DC5, DC6 and DC7 (Blechman et al., 2007). In this study, Vmat2:GFP transgenic zebrafish and the whole mount immunofluorescent staining were used to determine the location, size of dopaminergic nuclei during development, and the time to the development. Our results indicate that the neurons in the posterior nodule of the ventral DC, which may be the DC1/2 cell populations, were detected at 24 hpf. All the neuronal populations (DC1–7) in the ventral DC as well as those in LC, Ra, and telencephalon were detected at 48 hpf. Axons projected from the DC2 of TPp to telencephalon were detected at 3 dpf. The number of neuron in TPp increased from 24 hpf to 3 dpf and then decreased from 3 dpf to 5 dpf. It may be due to the apoptosis caused by failed connections between dopaminergic neurons and its targets which led to a lack of neurotrophic factors for them. These results provide some new experimental basis for further real time observation on the perikaryons, axons and survival of axon terminals of dopaminergic neurons, and for the study on the retrograde death of dopaminergic neurons induced by axonal degeneration.

In previous studies, the PD model was usually established in mice or rats, whose dopaminergic neuron location was better characterized. However, it is not very clear about the distribution of dopaminergic neurons in zebrafish. TH is the rate-limiting enzyme in the synthesis of DA and serves as a marker of dopaminergic and noradrenergic neurons (Kastenhuber et al., 2010; Tay et al., 2011). The experiments of Kaslin and Panula (2001), and Rink and Wullimann (2001) detected the distribution and axonal projections of dopaminergic neurons in adult zebrafish brain slices, respectively, using TH immunohistochemistry. Their results indicated that TH immune positive cells could be found in almost all the brain slices except for the mesencephalon, while the dopamine beta hydroxylase staining, representing the distribution of noradrenergic neurons, indicated that they were distributed only in deuterencephalon (Tay et al., 2011). Therefore the TH positive neurons in the prosencephalon of zebrafish were considered to be the dopaminergic neurons. In order to make a detailed study on the distribution of dopaminergic neurons in zebrafish, we evaluated the TH whole mount staining of WT zebrafish embryos at 4–6 dpf and compared it with that of the Vmat2:GFP transgenic zebrafish developed to the same period. We found that DC2 and DC4 neuronal populations were mainly dopaminergic neurons, while DC1, DC3, DC5 and DC6 might not, or could not be detected by staining due to their scarcity. Neurons in Pr and Vd/Vv were mainly dopaminergic, while neurons in LC and Ra might be noradrenergic.

The projection of dopaminergic neurons in zebrafish is closely related to aggression, addiction and control of some autonomous behaviors (Lam et al., 2005; Blechman et al., 2007). Thirumalai and Cline (2008) found that endogenous dopaminergic neurons could control the nerve conduction in swimming by presynaptic inhibition. However, as for the dyskinesia of zebrafish caused by neurotoxins (Fujimoto et al., 2011), the neuronal projections and the underlying mechanism are not clear. Our previous study found that, in the PD model induced by MPTP, the inhibition of dopaminergic neuron apoptosis could not prevent the initiation and progession of PD completely (Liang et al., 2008). The damage to the axonal microtubule system also played an important role in the degeneration and death of dopaminergic neurons (Wen et al., 2008). Therefore, it is important to study the axonal projection of dopaminergic neurons in zebrafish to establish the PD model and to investigate the pathogenesis of PD. The dopaminergic neurons of zebrafish locate mainly in the posterior nodule of the ventral DC (Kaslin and Panula, 2001). Their axons project very long and grew toward the median line region (Guo et al., 1999; Filippi et al., 2007; Ryu et al., 2007). These axons project to Pr. The preoptic area of the ventral DC and ventral telencephalon are connected by the anterior nerve tract (Guo et al., 1999; Boehmler et al., 2007). Present study suggested that the ventral DC of zebrafish is considered to be the substantia nigra of human, and the subpallium of zebrafish is considered to be the corpus striatum of human (Kawakami et al., 2000; Wullimann and Rink, 2002; Del Giacco et al., 2006). It is not known whether there is projection from the ventral DC to the subpallium in zebrafish, similar to the nigrostriatal pathway in human brain. In general, the axonal projections of catecholaminergic neurons have all grown out at 4 dpf (Smeets and González, 2000). So we used Vmat2:GFP transgenic zebrafish at 4 dpf to detect the projection. To our knowledge, we provided the first evidence that the axons from the TPp of the ventral DC projected to the subpallium *in vivo*. Although the existence of the above projections was confirmed, we are not sure whether this pathway is similar to the nigrostriatal pathway in human brain and whether it is related to the pathological changes of PD.

Our study systematically examined the developmental process of zebrafish dopaminergic neurons. DC2 and DC4 neuronal populations were detected at 24 hpf. Populations in LC, Ra and telencephalon were detected at 48 hpf. Axons were detected at 72 hpf, and we provided evidence that axons from the TPp of the ventral DC projected to the subpallium *in vivo*. However, when compared with results from whole mount TH immunofluorescence staining in WT zebrafish, we found that DC2 and DC4 neuronal populations were mainly dopaminergic, while DC1, DC3, DC5 and DC6 might not. Neurons in Pr and telencephalon were mainly dopaminergic, while neurons in LC and Ra might be noradrenergic. In conclusion, our study made some corrections and modifications on the localization and distribution of dopaminergic neurons in zebrafish. It provides further experimental evidences for the construction of the zebrafish PD model.

## Author Contributions

This study was based on the original idea of BS and SL. YD and QG contributed equally to this work. YD and QG observed the development and distribution of dopaminergic neurons of Vmat2:GFP embryos in real time and carried out the whole mount immunofluorescent staining. MS, YW, SH, HZ, HH, MY, XY, LR, JP, JS and HZ carried out the histology analysis and figures arrangement. BS drafted the manuscript and SL proofread the manuscript.

## Funding

This work was supported by National Nature Science Foundation of China (No: 31371215; 31540032), the Development and Regeneration Key Laboratory of Sichuan Province (SYS14-001; SYS15-002).

## Conflict of Interest Statement

The authors declare that the research was conducted in the absence of any commercial or financial relationships that could be construed as a potential conflict of interest.

## Abbreviations

DA: dopamine
DC: diencephalon
dpf: days post fertilization
Hc: caudal hypothalamus
hpf: hours post fertilization
LC: locus coeruleus
LSCM: laser scanning confocal microscope
MFB: midbrain/forebrain boundary
OB: olfactory bulb
Pa: paraventricular nucleus
PD: Parkinson’s disease
Pr: pretectum
PT: posterior tubercle
PTU: 1-phenyl 2-thiourea
PVN: hypothalamic paraventricular nucleus
PVO: paraventricular organ
Ra: raphe nuclei
TH: tyrosine hydroxylase
TPp: periventricular nucleus of posterior tubercle
Vd/Vv: dorsal/ventral nucleus of the telencephalic area
WT: wild type.

## References

[B1] Blechman J. Borodovsky N. Eisenberg M. Nabel-Rosen H. Grimm J. Levkowitz G. (2007). Specification of hypothalamic neurons by dual regulation of the homeodomain protein orthopedia. Development 134, 4417–4426. doi: 10.1242/dev.011262 18003738 10.1242/dev.011262

[B2] Boehmler W. Carr T. Thisse C. Thisse B. Canfield V. A. Levenson R. (2007). D4 Dopamine receptor genes of zebrafish and effects of the antipsychotic clozapine on larval swimming behaviour. Genes Brain Behav. 6, 155–166. doi: 10.1111/j.1601-183x.2006.00243.x 16764679 10.1111/j.1601-183X.2006.00243.x

[B3] Del Giacco L. Sordino P. Pistocchi A. Andreakis N. Tarallo R. Di Benedetto B.. (2006). Differential regulation of the zebrafish *orthopedia 1* gene during fate determination of diencephalic neurons. BMC Dev. Biol. 6:50. doi: 10.1186/1471-213X-6-50 17074092 10.1186/1471-213X-6-50 PMC1635040

[B4] Filippi A. Dürr K. Ryu S. Willaredt M. Holzschuh J. Driever W. (2007). Expression and function of *nr4a2*, *lmx1b,* and *pitx3* in zebrafish dopaminergic and noradrenergic neuronal development. BMC Dev. Biol. 7:135. doi: 10.1186/1471-213x-7-135 18053265 10.1186/1471-213X-7-135 PMC2217549

[B5] Fujimoto E. Stevenson T. J. Chien C. B. Bonkowsky J. L. (2011). Identification of a dopaminergic enhancer indicates complexity in vertebrate dopamine neuron phenotype specification. Dev. Biol. 352, 393–404. doi: 10.1016/j.ydbio.2011.01.023 21276790 10.1016/j.ydbio.2011.01.023 PMC3069253

[B6] Guo S. Wilson S. W. Cooke S. Chitnis A. B. Driever W. Rosenthal A. (1999). Mutations in the zebrafish unmask shared regulatory pathways controlling the development of catecholaminergic neurons. Dev. Biol. 208, 473–487. doi: 10.1006/dbio.1999.9204 10191060 10.1006/dbio.1999.9204

[B7] Kaslin J. Panula P. (2001). Comparative anatomy of the histaminergic and other aminergic systems in zebrafish *(Danio rerio)*. J. Comp. Neurol. 440, 342–377. doi: 10.1002/cne.1390 11745628 10.1002/cne.1390

[B8] Kastenhuber E. Kratochwil C. F. Ryu S. Schweitzer J. Driever W. (2010). Genetic dissection of dopaminergic and noradrenergic contributions to catecholaminergic tracts in early larval zebrafish. J. Comp. Neurol. 518, 439–458. doi: 10.1002/cne.22214 20017210 10.1002/cne.22214 PMC2841826

[B9] Kawakami K. Shima A. Kawakami N. (2000). Identification of a functional transposase of the *Tol2* element, an *Ac*-like element from the Japanese medaka fish and its transposition in the zebrafish germ lineage. Proc. Natl. Acad. Sci. U S A 97, 11403–11408. doi: 10.1073/pnas.97.21.11403 11027340 10.1073/pnas.97.21.11403 PMC17212

[B10] Lam C. S. Korzh V. Strahle U. (2005). Zebrafish embryos are susceptible to the dopaminergic neurotoxin MPTP. Eur. J. Neurosci. 21, 1758–1762. doi: 10.1111/j.1460-9568.2005.03988.x 15845104 10.1111/j.1460-9568.2005.03988.x

[B11] Liang Y. Li S. Wen C. Zhang Y. Guo Q. Wang H.. (2008). Intrastriatal injection of colchicine induces striatonigral degeneration in mice. J. Neurochem. 106, 1815–1827. doi: 10.1111/j.1471-4159.2008.05526.x 18564367 10.1111/j.1471-4159.2008.05526.x

[B12] Ma P. M. (1994). Catecholaminergic systems in the zebrafish. II. Projection pathways and pattern of termination of the locus coeruleus. J. Comp. Neurol. 344, 256–269. doi: 10.1002/cne.903440207 8077460 10.1002/cne.903440207

[B13] Ma P. M. (1997). Catecholaminergic systems in the zebrafish. III. Organization and projection pattern of medullary dopaminergic and noradrenergic neurons. J. Comp. Neurol. 381, 411–427. doi: 10.1002/(SICI)1096-9861(19970519)381:4<411::AID-CNE2>3.0.CO;2-5 9136799

[B14] Rink E. Wullimann M. F. (2001). The teleostean (zebrafish) dopaminergic system ascending to the subpallium (striatum) is located in the basal diencephalon (posterior tuberculum). Brain Res. 889, 316–330. doi: 10.1016/s0006-8993(00)03174-7 11166725 10.1016/s0006-8993(00)03174-7

[B15] Riparbelli M. G. Callaini G. (2007). The *Drosophila parkin* homologue is required for normal mitochondrial dynamics during spermiogenesis. Dev. Biol. 303, 108–120. doi: 10.1016/j.ydbio.2006.10.038 17123504 10.1016/j.ydbio.2006.10.038

[B16] Ryu S. Mahler J. Acampora D. Holzschuh J. Erhardt S. Omodei D.. (2007). Orthopedia homeodomain protein is essential for diencephalic dopaminergic neuron development. Curr. Biol. 17, 873–880. doi: 10.1016/j.cub.2007.04.003 17481897 10.1016/j.cub.2007.04.003

[B17] Smeets W. J. A. J. González A. (2000). Catecholamine systems in the brain of vertebrates: new perspectives through a comparative approach. Brain Res. Rev. 33, 308–379. doi: 10.1016/s0165-0173(00)00034-5 11011071 10.1016/s0165-0173(00)00034-5

[B18] Tay T. L. Ronneberger O. Ryu S. Nitschke R. Driever W. (2011). Comprehensive catecholaminergic projectome analysis reveals single-neuron integration of zebrafish ascending and descending dopaminergic systems. Nat. Commun. 2:171. doi: 10.1038/ncomms1171 21266970 10.1038/ncomms1171 PMC3105308

[B19] Thirumalai V. Cline H. T. (2008). Endogenous dopamine suppresses initiation of swimming in prefeeding zebrafish larvae. J. Neurophysiol. 100, 1635–1648. doi: 10.1152/jn.90568.2008 18562547 10.1152/jn.90568.2008 PMC2544474

[B20] Wen L. Wei W. Gu W. Huang P. Ren X. Zhang Z.. (2008). Visualization of monoaminergic neurons and neurotoxicity of MPTP in live transgenic zebrafish. Dev. Biol. 314, 84–92. doi: 10.1016/j.ydbio.2007.11.012 18164283 10.1016/j.ydbio.2007.11.012

[B21] Wullimann M. F. Rink E. (2002). The teleostean forebrain: a comparative and developmental view based on early proliferation, Pax6 activity and catecholaminergic organization. Brain Res. Bull. 57, 363–370. doi: 10.1016/s0361-9230(01)00666-9 11922990 10.1016/s0361-9230(01)00666-9

[B22] Yang Y. Ouyang Y. Yang L. Beal M. F. McQuibban A. Vogel H.. (2008). Pink1 regulates mitochondrial dynamics through interaction with the fission/fusion machinery. Proc. Natl. Acad. Sci. U S A 105, 7070–7075. doi: 10.1073/pnas.0711845105 18443288 10.1073/pnas.0711845105 PMC2383971

